# Predicting developmental dysplasia of the hip in at-risk newborns

**DOI:** 10.1186/s12891-020-03454-4

**Published:** 2020-07-07

**Authors:** Andreas Roposch, Evangelia Protopapa, Olivia Malaga-Shaw, Yael Gelfer, Paul Humphries, Deborah Ridout, John H. Wedge

**Affiliations:** 1 Institute of Child Health, University College London, 30 Guildford St, London, WC1N 3EH UK; 2 Department of Orthopaedic Surgery, Great Ormond Street Hospital for Children, London, UK; 3 Royal Free Hospital NHS Trust, London, UK; 4 Department of Orthopaedic Surgery, St George’s Hospital, London, UK; 5 Department of Diagnostic Imaging, University College Hospital, London, UK; 6 Department of Surgery, University of Toronto, Toronto, Canada

**Keywords:** Developmental dysplasia of the hip, Risk factors, New born, Prediction model

## Abstract

**Background:**

The development of developmental dysplasia of the hip can be attributed to several risk factors and often in combination with each other. When predicting the likelihood of developing this condition, clinicians tend to over and underestimate its likelihood of occurring.

Therefore, the study aim is to determine among at-risk newborns how to best predict developmental dysplasia of the hip (DDH) within 8 weeks post-partum.

**Methods:**

Prospective cohort study in secondary care. Patient population included newborns at-risk for DDH – we assessed 13,276 consecutive newborns for the presence of DDH risk factors. Only newborns with at least one of the predefined risk factors and those showing an abnormal examination of the hip were enrolled (*n* = 2191). For the development of a risk prediction model we considered 9 candidate predictors and other variables readily available at childbirth.

The main outcome measure was ultrasonography at a median age of 8 weeks using consensus diagnostic criteria; outcome assessors were blinded.

**Results:**

The risk model includes four predictors: female sex (OR = 5.6; 95% CI: 2.9–10.9; *P* <  0.001); first degree family history of DDH (OR = 4.5; 95% CI: 2.3–9.0; *P* <  0.001), birthweight > 4000 g (OR = 1.6; 95% CI: 0.6–4.2; *P* = 0.34), and abnormal examination of hip (OR = 58.8; 95% CI: 31.9, 108.5; *P* <  0.001). This model demonstrated excellent discrimination (C statistic = 0.9) and calibration of observed and predicted risk (*P* = 0.35). A model without the variable ‘hip examination’ demonstrated similar performance.

**Conclusion:**

The risk model quantifies absolute risk of DDH within 8 weeks postpartum in at-risk newborns. Based on clinical variables readily available at the point of childbirth, the model will enhance parental counselling and could serve as the basis for real time decisions prior to discharge from maternity wards.

## Background

In early infancy the spectrum of developmental dysplasia of the hip (DDH) represents a continuum of risk from benign self-limiting instability to frank dislocation that requires rapid orthopaedic input. Multiple widely accepted perinatal risk factors are used to identify *at-risk newborns* and triage them to, for example, rapid orthopaedics referral or selective ultrasound screening. Newborns and infants with the following features are generally thought to be at-risk for DDH: female sex, family history of DDH, breech delivery, first born, and abnormal examination of the hip [1]. Less widely accepted factors include foot deformities, [2, 3] high birth weight, [4] and torticollis [5].

With use of these factors clinicians on maternity wards estimate the likelihood of DDH to be present or to develop; counsel parents of affected newborns; and make triage decisions. However, the risk factors often tend to occur in combinations and since they are interrelated, clinicians could over or underestimate the likelihood of DDH being present or becoming present. A risk model that could help clinicians making predictions about DDH for individual at-risk newborns would thus be a useful tool for clinicians.

The aim of our study was to develop a new prediction model that can generate absolute predicted risk of DDH within 8 weeks postpartum on the basis of each at-risk newborn’s individualised clinical risk profile.

## Methods

### Setting and eligibility

Consecutive newborns of an urban teaching hospital in London, England, and those referred by family physicians were eligible for this prospective cohort study, conducted from November 2010 to January 2013. This hospital provides ante and postnatal services for the local population and acts as a tertiary referral unit for pregnant women and neonates. The local population is mixed representing all socioeconomic groups in a multi ethnic community. Newborns were ineligible if they had a syndrome or neurological disorder, which by definition excludes DDH as a possible diagnosis. The Institutional Review Board approved the study. Caregivers provided written informed consent.

### Predictor selection and measurement

A dedicated research team examined each newborn and community referral and ascertained all variables prospectively. The median newborn age at this assessment was 1 day (interquartile range, 0–1 days). We assessed newborns for the presence of widely-accepted DDH risk factors, based on systematic reviews, [1, 5, 6] clinical guidelines, [7] and a consensus document of experts from 34 countries [8]. These included a family history of DDH in a first degree relative (parental self-report); breech lie in the third trimester with cephalic presentation or breech presentation at birth; and history of oligohydramnios (ultrasound-based diagnosis at 18th to 20th week of gestation with amniotic fluid index ≤5). We also recorded sex, birth weight; whether it was a first born child; a twin pregnancy; a vaginal or a caesarean delivery.

All newborns underwent an examination of their hips and we ascertained the presence of standardized diagnostic criteria for DDH [8] – Ortolani or Barlow sign; asymmetry in hip abduction ≥20^°^; leg length discrepancy (Galeazzi sign). We recorded the presence of a torticollis. We included all foot deformity and grouped them (based on the assessment of a physiotherapist) into those with marked postural deformities (e.g. metatarsus adductus; calcaneovalgus) that we followed on at least one occasion with a physiotherapist to ensure improvement; and those with clubfeet that we immediately referred to a surgeon). Neonatology residents, overseen by attending neonatologists and dedicated research nurses, carried out these assessments. Newborns with abnormal or equivocal clinical examination of the hip underwent a concurrent examination by an attending neonatologist or attending pediatric orthopaedics surgeon to confirm eligibility. During the course of the study 68 infants were referred by family physicians for a possible diagnosis of DDH due to risk factors and we included them all. Central project staff ensured quality through training sessions and regular on-site data quality audits. We ensured recruitment of consecutive patients by daily comparing the number of newborns with the number of case report forms completed (birth occurring on weekends were compared on Mondays). The same was done weekly for referrals made by family physicians. We randomly performed double examination of 230 hips (115 infants) to determine the inter-rater reliability in the hip examination between the team of residents and a pediatric orthopaedics surgeon: hips were examined reliably (inter-rater κ = 0.90).

### Outcome

One outcome was assessed for all patients – ultrasound-based diagnosis of DDH at age 6–8 weeks (median, 8 weeks). We classified sonograms with use of standardized diagnostic criteria based on international consensus [8] which define an α-angle [9] < 55° as DDH. This outcome is robust as any such finding warrants monitoring or treatment [8]. In rare instances where hips were scanned before 6 weeks because of suspected dislocation, we defined α < 43° as DDH [8].

Two senior attending pediatric radiologists and 3 sonographers specifically trained in infant hip ultrasonography performed (and reported) all ultrasound tests in dedicated clinics according to a standardized protocol [9] (GE Medical Systems, Chalfont St. Giles, United Kingdom). We (AR, PH) evaluated all scans that had been reported as α < 60°, blinded to predictors and original report; we performed our own measurement of the α-angle, which we used for this study. We did the same for a random sample of scans that had been reported as α > 60°; we found that our measurements were consistent with the initial report in all cases and thus assumed robustness of the initial report. Inter-rater reliability studies for the α-angles in the original scan reports and that reported by the study team showed an intra-class correlation coefficient of 0.88 (95% confidence interval, 0.86–0.90).

**Fig. 1:**
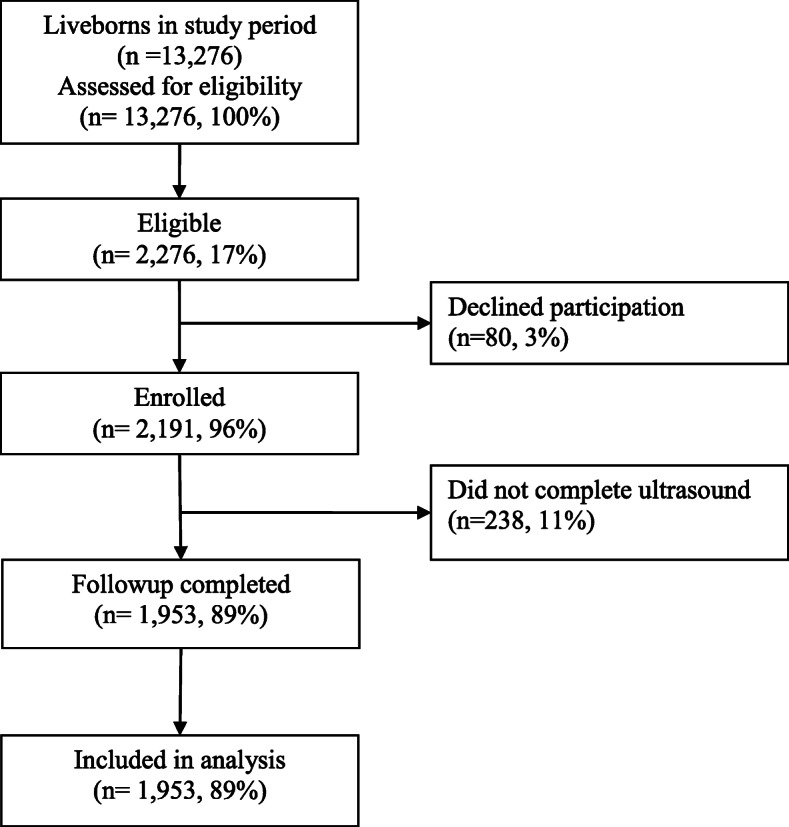
Flowchart showing patient eligibility and enrollment

Of 13,208 livebirths and 68 community referrals, 2276 (17%) newborns were eligible (Fig. 1). Of those, 80 (3%) caregivers declined participation. Of 2191 consented newborns, 1953 (89%) completed the ultrasound and were included in the analysis; 238 (11%) infants who did not were excluded (Table 1).

**Table 1:** Baseline characteristics of 2271 eligible infants. Values are numbers (percentages) unless stated otherwise

**Variable**	**Enrolled** **(*N*** **= 2191)**	**Declined participation** **(*N*** **= 80)**
Recruited from maternity ward	2123 (96.9)	76 (95)
Referred by family physician	68 (3.1)	4 (5)
Mean age at assessment in days (SD)	3.5 (14.8)	2.1 (5.6)
Lost to follow-up	220 (10)	4 (5)
Received treatment for DDH	76 (3.5)	3 (4)
Female-to-male ratio	1142:1049	44:36
Female sex	1142 (52.1)	44 (55)
First born child	1229 (56.1)	unknown
One of twins (twin pregnancy)	285 (13)	6 (8)
Birth weight in grams (mean ± SD)	3220 ± 583	unknown
Oligohydramnios	50 (2.3)	2 (3)
Breech lie in last trimester or at presentation	1419 (64.8)	45 (56)
Breech presentation	640 (29.2)	18 (23)
Breech lie in last trimester with cephalic presentation	779 (35.6)	27 (34)
Vaginal delivery	1020 (46.6)	unknown
Caesarean section	1131 (51.6)	unknown
First degree family history of DDH	205 (9.4)	3 (4)
Torticollis	12 (0.5)	0 (0)
Foot deformity with orthopaedic follow-up	9 (0.4)	2 (3)
Foot deformity with physiotherapist follow-up	46 (2.1)	3 (4)
Leg length discrepancy (Galeazzi sign)	16 (0.7)	1 (1)
Asymmetry in hip abduction	39 (1.8)	1 (1)
Barlow positive	21 (1)	0 (1)
Ortolani positive	14 (0.6)	1 (1)
Hip instability	27 (1.2)	1 (1)

*SD* standard deviation

### Statistical analysis

We summarized all variables as means and standard deviations or frequencies and proportions, respectively. We treated the occurrence of DDH as a binary outcome (based on α < 55° at age > 42 days, or α < 50 at age < 42 days). We considered for inclusion in the risk prediction model only candidate predictors with a prevalence of > 2% in order to avoid imprecise estimates of regression coefficients [10]. We combined less frequently occurring predictors if clinically plausible (e.g. we created the variable *abnormal hip examination*, encompassing any hips showing any of the following: positive Barlow, Ortolani, or Galeazzi signs, or asymmetry in abduction ≥20^°^ - hips exhibiting multiple of these criteria were counted once only). We also combined all foot deformities warranting followup with a physiotherapist or with a surgeon into one variable, ‘foot deformity’. The variables oligohydramnios and torticollis were omitted from analysis due to their low prevalence. This resulted in 9 candidate predictors to be analyzed (Additional file 1).

Variables associated with DDH at *p* <  0.10 in simple logistic regression were taken to multivariate analysis. They were: female sex, family history of DDH, first born, birth weight, foot deformity warranting followup, and abnormal hip examination. Because “breech presentation/breech in last trimester” is a widely accepted risk factor, we explored this variable further despite it not meeting this threshold. We tested if the mode of delivery (vaginal or caesarean) would affect its association with DDH. No such effect was seen. Hence, we omitted ‘breech presentation/breech in last trimester’ from further analyses. We dichotomised the variable birth weight (because it would be easier to use for clinicians) based on an accepted threshold of 4000 g [4, 8].

For derivation of a risk prediction model, we included all 6 candidate predictors in a multivariable logistic regression model and used backward elimination and retained predictors at the 5% significance level. Four of 6 predictors retained in the model: female sex; family history; birth weight > 4000 g; and abnormal hip examination. We also developed a model without the variable ‘abnormal hip examination’ as this may be of interest for certain groups of clinicians.

Birthweight was missing for 61 cases and first born was missing for 35 cases. We used multiple imputation (assuming data were missing at random) to account for this missing data when fitting the final multivariable models. The imputation models included all risk factors considered in the univariate analysis (which we assume includes all predictors of missingness). We generated 20 data sets and ran logistic regression, using the whole data set and implementing a bootstrap (200 samples) for each imputed data set to correct for overfitting. Results from analyses using 20 imputed datasets were compared with those only including infants without missing data and no differences were found. We derived the final models by fitting a logistic regression model for all significant predictors. Estimates were combined using Rubin rules [11].

Outcome data was missing for 238 infants. Summaries of risk factor data showed these cases were not different to cases with available ultrasound information. Furthermore, we ran a sensitivity analysis assuming all these 238 cases did not have DDH and found that results of univariate analyses were similar to those for 1953 cases; hence we ran multivariable models for cases with available outcome data only.

We assessed the performance of the model in terms of the C statistic (a value of 1.0 is perfect) and calibration. The C statistic represents the probability that for any randomly selected pair of newborns with and without DDH, the newborn who had DDH had a higher predicted risk. Calibration refers to how similar the model-estimated likelihood of DDH was to the observed likelihood of DDH (Hosmer–Lemeshow goodness-of-fit test – a well-fitting model will result in *p* > 0.05).

We validated the model to correct measures of predictive performance for optimism by bootstrapping 200 samples of the derivation data. We repeated the model development process in each bootstrap sample as outlined above in order to produce a model, we applied the model to the same bootstrap sample to quantify apparent performance, and we applied the model to the original dataset to test model performance (C statistic and calibration) and optimism (difference in test performance and apparent performance). We then estimated the overall optimism across all models.

Sample size — in planning the study, we anticipated to evaluate 6–7 independent covariates with a high-enough (> 2%) prevalence in our sample. Our study revealed 77 cases of DDH, which allows for 8 covariates to be examined in multivariable analysis [12].

The local research ethics board approved this study (research ethics committee reference: 14/LO/0420).

## Results

Of 9 candidate predictors examined, 6 were associated (*P* < 0.1) with DDH in univariate analysis (Table 2). Of these, we ultimately retained 4 in the risk prediction model: female sex (OR = 5.6, 95% CI, 2.9–10.9; *P* < 0.001); family history of DDH (OR = 4.5, 95% CI, 2.3–9.0; *P* < 0.001); birthweight > 4000 g (OR = 1.6, 95% CI, 0.6–4.2; *P* = 0.34); abnormal examination of hip (OR = 58.8, 95% CI, 31.9–108.5; *P* < 0.001) (Table 3). This model discriminated well between newborns with and without DDH (C statistic = 0.9, 95% CI, 0.8–0.9; goodness-of-fit *P* = 0.35). Omitting 68 community referrals from the analysis did not change these results.

**Table 2:** Simple regression analysis of candidate predictors in 1953 infants who had outcomes ascertained (DDH was defined as α < 55° at age > 42 days, or α < 50 at age < 42 days). All values are numbers (percentages) unless otherwise stated

**Candidate Predictor**	**All** **infants** **(*N*= 1953)**	**Infants with DDH** **(*N* = 77)**	**Odds ratio** **(95% confidence interval)**	***P***
Female sex	1037 (53.1)	65 (77)	5.04 (2.70, 9.39)	< 0.001
First degree family history of DDH	184 (9.4)	15 (9)	2.44 (1.36, 4.39)	0.003
Vaginal delivery	892 (45.7)	42(58)	1.59 (0.99, 2.55)	0.06
Birth weight in kg (mean ± SD)	3.22 ± 0.58	3.40 ± 0.52	1.81 (1.14, 2.87)	0.01
Foot deformity warranting followup ^c^	51 (2.6)	5 (8)	2.76 (1.07, 7.16)	0.04
First born child	1108 (56.7)	46 (66)	1.35 (0.82, 2.23)	0.24
Multiple birth (twin pregnancy)	257 (13.2)	6 (9)	0.61 (0.26, 1.41)	0.25
Breech at presentation	586 (30.0)	20 (39)	0.81 (0.48, 1.36)	0.43
Oligohydramnios	39 (2.0)	0 (0)	NA^a^	NA^a^
Torticollis	10 (0.5)	1 (1)	NA^b^	NA^b^
Abnormal hip examination ^d^	86 (4.4)	43 (56)	53.91 (31.35, 92.71)	< 0.001
Ortolani positive	14 (0.7)	11 (14)	NA^b^	NA^b^
Barlow positive	20 (1.0)	14 (18)	NA^b^	NA^b^
Galeazzi positive (leg length discrepancy)	16 (0.8)	11 (14)	NA^b^	NA^b^
Hip instability	25 (1.3)	7 (9)	NA^b^	NA^b^
Asymmetry in hip abduction	36 (1.8)	21 (27)	NA^b^	NA^b^

*SD* standard deviation

^a^Not possible to estimate because no DDH cases had oligohydramnios

^b^Not applicable (NA) for risk model due to low prevalence

^c^Foot deformity receiving orthopedic or physiotherapy follow-up

^d^This variable includes hips with at least one of the following criteria present: leg length discrepancy, asymmetry of hip abduction, Barlow positive, Ortolani positive, hip instability. The latter 3 were mutually exclusive. This variable thus indicates hips that showed an abnormal physical examination

**Table 3:** Risk prediction model

Risk score from a logistic regression model to predict DDH in the first eight weeks postpartum. Risk score = − 5.50 + 1.73*female sex* + 1.51*first degree family history* + 4.07*hip-related factors* + 0.48*infant birth weight > 4000 g.* All variables are coded as binary (0 or 1 for absence or presence of a risk factor), except for infant birth weight. The value − 5.50 is the intercept, and other numbers are the estimated regression coefficients for the predictors, which indicate their mutually adjusted relative contribution to the outcome risk. The regression coefficients represent the log odds ratio for a change of 1 unit in the corresponding predictor. The predicted risk of DDH = 1/1 + e^−riskscore^.	
*Example 1*—A newborn whose older sibling received splinting for DDH has a birth weight of 4100 g. She showed no abnormalities on the newborn physical examination, in particular her hips were stable and showed symmetric range of motion. She has a predicted risk of 14% of developing DDH within the first eight weeks of delivery. *Interpretation*: if 100 newborns with the same risk factors are followed, one will develop DDH within eight weeks of birth.	
*Example 2*—A newborn girl was examined on the second day post-partum before discharge from the maternity unit. The left hip was restricted in movement, in particular in abduction. It appeared that the leg lengths were different. The child’s predicted DDH risk is 86% within the first eight weeks of delivery. *Interpretation*: if 100 newborns with the same risk factors are followed, ninety will develop DDH within eight weeks of birth	

A model omitting the variable ‘abnormal hip examination’ gave the following odds ratios: female sex 5.8 (95% CI, 3.1–11.0; *P* < 0.001), family history of DDH 2.6 (95% CI, 1.4–4.7; *P* < 0.001), birthweight > 4000 g 3.1 (95% CI, 1.6–6.0; *P* < 0.001). This model also discriminated between cases with and without DDH (C statistic 0.7, 95% CI, 0.7–0.8; goodness-of-fit *P* = 0.76).

## Discussion

Multiple widely-accepted risk factors obtained at bedside are used to identify newborns at-risk for DDH. The additive effects and the interrelationships of these risk factors, however, remain ill-defined. Understanding how to best harness the several risk factors in order to calculate an individual newborn’s risk of being diagnosed with DDH within 8 weeks of birth could be an important addition to the counselling of mothers of at-risk infants, and to the design of postnatal care pathways. We present a new risk prediction model to calculate the absolute risk of DDH in the first 8 weeks postpartum in a large representative sample of at-risk newborns.

### Model performance

Our prediction model demonstrated excellent discrimination, that is, the ability to predict correctly amongst at-risk newborns those who have DDH. And it does so with use of only 4 perinatal risk factors. To correct measures of predictive performance for optimism, we did internal validation by bootstrapping. The optimism-adjusted C statistic was 0.9, with a lower bound of 0.8 in its 95% confidence interval, indicating excellent discrimination. We observed good agreement of DDH cases based on our model’s prediction and the original data: only 2 of 77 (2.5%) newborns with DDH were not predicted by our model.

Based on our model at-risk newborns have a 19% risk of obtaining a diagnosis of DDH within 8 weeks if the newborn hip examination is abnormal. This risk is markedly lower for newborns who exhibit other single risk factors, ranging from 0.4% for those with a birthweight > 4000 g to 2% for those who are girls or those who have a positive family history. The model revealed the nature of the additive effect of risk factors – the risk is 50% if an abnormal hip exam is found in newborns with a family history of DDH; or 67% if found in girls. A very high risk of 86% occurs in girls who have an abnormal hip exam as well as a positive family history (Table 3).

### Model content

The strongest single predictor was an abnormal physical examination of the hip – 43 of 86 (50%) infants with an abnormal examination of the hip had DDH confirmed. An abnormal examination of the hip is not always a definite symptom of DDH since hips can improve spontaneously in this age group. However, the second model that we present omits the hip examination entirely and it performed comparably well in predicting cases with and without DDH. We also encountered 34 cases of DDH who exhibited a normal examination of the hip and our model is particularly useful for this group.

Whilst we expected the variable ‘breech during last trimester/breech delivery’ to be part of the risk prediction model, it was not. Its odds ratio was 1.1 in an analysis adjusted for the mode of delivery, with a 95% confidence interval from 0.58 to 2.07 [13]. However, the reported association between breech delivery and DDH varies widely, with risk ratios for comparable age groups ranging from ≥8.0 [4, 14] to 1.1 [15, 16] to no association [17, 18]. Perhaps the sub-type ‘extended breech’ would have shown statistical significance but since we did not measured this detail this remains speculative. Whether a foot deformity is a risk factor for DDH remains controversial [2, 19] and our study was not designed to clarify this issue. The definition of foot deformities in newborns can be subjective, [14] with the potential for measurement bias. We thus regarded only foot deformities that required orthopaedics or physiotherapy followup (these were severe metatarsus adductus and clubfeet) for the analysis. In line with previous research, [19] having a foot deformity was not associated with DDH in our study.

The ascertainment of the variable ‘physical examination’ was of particular importance. Because we wanted to create a risk prediction tool for use by maternity ward doctors, we deliberately had neonatology residents examine the hips of study participants. Although orthopaedics surgeons or neonatologists may generally be better skilled in this task, they are typically not the ones who carry out this task in daily clinical care. In order to ensure robust collection of this information, we conducted double-examinations of all newborns whose hips were deemed to be abnormal by the residents. Further, throughout the study period, paediatric orthopaedics surgeons periodically trained all residents in the clinical examination of the newborn hip. Whilst it is not impossible that some abnormal physical examination findings still went unnoticed, our model performance reflects the average skills of those who are conducting the postnatal examinations, which strengthens its generalizability.

### Strengths and limitations

We developed our prediction model with a high degree of precision. In assembling the cohort, we defined ‘at-risk’ with use of widely-accepted perinatal risk factors based on a metaanalysis [1], an international Delphi consensus study [8], and a population-based cohort study [4]. We defined DDH by means of diagnostic criteria based on the consensus of international experts [8]. We measured outcome and predictors with great rigor using double examinations, consensus readings, and blinding where possible. We enrolled consecutive newborns and observed high accrual.

We note the limitations of this study. First, the variables ‘torticollis’ and ‘oligohydramnios’ were not encountered frequently enough allow for statistical analysis. Larger studies are needed to clarify if these two variables improve the predictive performance of the model. Second, the mothers of 3.5% of eligible newborns declined participation. However, there is no evidence of sampling bias; enrolled newborns were largely similar in their risk factors as those enrolled (Table 1). Third, the outcome was not ascertained by the study team in 238 (11%) of enrolled cases. Of these, 24 had it ascertained elsewhere with 2 cases of DDH confirmed; and 76 never had an ultrasound scan done at all. It remains unknown what happened to further 144 enrolled cases. We explored the distribution of predictors in those with and without ultrasound outcomes and they were similar. We then did a sensitivity analysis of 2191 participants whereby we assumed all the unknown outcomes to be non-DDH; the results were similar to the analysis of 1953 participants who had the ultrasound done by the study team.

## Conclusions

Our prediction model can be used to express the absolute predicted risk of DDH within 8 weeks postpartum for an individual at-risk newborn. The model is based on clinical variables available at the point of childbirth. For newborns with one or more risk factors for DDH, our model enables clinicians to refine the risks associated with each combination of risk factors. This information could enhance the counselling of caregivers. It could also be used to refine existing care pathways for at-risk newborns in terms of ‘levels of urgency’ for in the postnatal period. For example, those with risks > 80% could be booked directly and before discharge from maternity wards for a two-week orthopaedics surgeon consultation, and those with lower risks could be given ultrasound appointments at 6 weeks, etc. Naturally, such decision will need to be based on local resources and are beyond the scope of this study.

## Supplementary information

**Additional file 1. **Flowchart showing the development of the risk prediction model. We considered to include “breech presentation” in the model – despite it was not significant in univariate analysis – and tested the effect of breech in a model adjusted for mode of delivery. This effect was not significant (OR=1.10, *p* = 0.76) and we omitted breech from further analysis.

## Data Availability

The datasets generated and/or analysed during the current study are not publicly available due to data protection/potential risk to identify study participants but are available from the corresponding author on reasonable request.

## Abbreviation

DDH: Developmental dysplasia of the hip
