# Supplementary material for: Predicting developmental dysplasia of the hip in at-risk newborns
Source: BMC Musculoskelet Disord. 2020 Jul 7;21:442. doi: 10.1186/s12891-020-03454-4 (PMC7341560; doi:10.1186/s12891-020-03454-4)
Supplement: Supplementary file 1 — Additional file 1. Flowchart showing the development of the risk prediction model. We considered to include “breech presentation” in the model – despite it was not significant in univariate analysis – and tested the effect of breech in a model adjusted for mode of delivery. This effect was not significant (OR=1.10, p = 0.76) and we omitted breech from further analysis. [file 12891_2020_3454_MOESM1_ESM.docx]

**Appendix 1.** Flowchart showing the development of the risk prediction model. We considered to include “breech presentation” in the model – despite it was not significant in univariate analysis – and tested the effect of breech in a model adjusted for mode of delivery. This effect was not significant (OR=1.10, p= 0.76) and we omitted breech from further analysis.

11 candidate predictors were collected in the cohort study, of which only 9 (numbers 1-9 below) could be examined due to sufficiently high prevalence.

Multivariate analysis retained 4 of the 6 initial predictors. This model showed good discrimination (*C statistic*=0.88) and good calibration (*Hosmer–Lemeshow* *test* p=0.35).

Univariate analysis showed that 6 were associated (p<0.1) with DDH. Interaction breech*delivery mode was not observed. These 6 were taken to next step.

| **1** | **Female sex** |  | **Female sex** |  | **Female sex** | OR=5.62 |
| --- | --- | --- | --- | --- | --- | --- |
| **2** | **First degree family history of DDH** |  | **First degree family history of DDH** |  | **First degree family history of DDH** | OR=4.53 |
| **3** | **Vaginal delivery** |  | **Vaginal delivery** |  | **Birth weight >4000g** | OR=1.61 |
| **4** | **Breech presentation** |  | **Birth weight >4000g** |  | **Abnormal hip examination** | OR=58.78 |
| **5** | **Twin pregnancy** |  | **Foot deformity warranting followup** |  |  |  |
| **6** | **First born child** |  | **Abnormal hip examination** |  |  |  |
| **7** | **Birth weight in kg** |  |  |  |  |  |
| **8** | **Foot deformity warranting followup** |  |  |  |  |  |
| **9** | **Abnormal hip examination** |  |  |  |  |  |
| 10 | Torticollis |  |  |  |  |  |
| 11 | Oligohydramnios |  |  |  |  |  |
